# Usefulness and Reliability of the Bispectral Index during Balanced Anesthesia for Neurovascular Surgery in New Zealand White Rabbits

**DOI:** 10.3390/brainsci13020327

**Published:** 2023-02-14

**Authors:** Mariafrancesca Petrucci, Claudia Spadavecchia, Stefan Wanderer, Gwendoline Boillat, Serge Marbacher, Luisana Gisela García Casalta, Daniela Casoni

**Affiliations:** 1 Experimental Surgery Facility (ESF), Department for BioMedical Research, Faculty of Medicine, University of Bern, 3010 Bern, Switzerland; 2 Anesthesiology and Pain Therapy Section, Department of Clinical Veterinary Medicine, Vetsuisse Faculty, University of Bern, 3012 Bern, Switzerland; 3 Department of Neurosurgery, Kantonsspital Aarau, 5001 Aarau, Switzerland; 4 Cerebrovascular Research Group, Department for BioMedical Research, University of Bern, 3010 Bern, Switzerland

**Keywords:** bispectral index, balanced anesthesia, neuromonitoring, neurosurgery, rabbits, fentanyl, carotid bifurcation, experimental anesthesia

## Abstract

Few data about the electroencephalogram and its calculated indices, such as the bispectral index (BIS), have been reported in rabbits. We aimed to evaluate whether a clinically stable anesthesia was mirrored by consistent and stable BIS values and to investigate the effects of modified cerebral blood supply, due to bilateral carotid clamping and re-opening, on BIS values. We also investigated the effects of fentanyl, as an antinociceptive drug, on the BIS. Sixty-eight rabbits undergoing general anesthesia for surgical creation of carotid bifurcation aneurysms were enrolled. The BIS values were recorded at nine selected time points (TPs) during each procedure and before and after fentanyl administration. The BIS values over time were compared with two-way repeated-measures analysis of variance followed by Tukey test, while the Wilcoxon signed rank test was performed to compare values at clamping and re-opening of the carotids as well as before and after fentanyl administration. The BIS values were significantly lower during anesthesia than at the end of anesthesia and at tracheal extubation; no significant differences were found among other TPs. Adequate depth of anesthesia was mirrored by consistent BIS values among rabbits, and alteration of cerebral blood supply did not modify BIS values, except once. Following fentanyl, BIS values did not change in a clinically relevant way.

## 1. Introduction

Depth of anesthesia is a dynamic condition which depends on both degree of cortical depression, that is reversibly caused by anesthetic agents, and nociceptive inhibition [1]. Its accurate assessment is challenging, although of paramount importance for allowing anesthetists to tailor drugs’ administration, prevent excessive depth or accidental awareness, and improve patient outcome [2]. Depth of anesthesia is generally assessed through the evaluation of reflexes, autonomic responses, and presence or absence of movements following nociceptive stimulations. Depth of anesthesia indexes, based on electroencephalographic (EEG) activity, have also been used for this purpose [3,4]. Among them, the most investigated has been the bispectral index (BIS), an index produced by an algorithm based on bispectral, power spectral, and time domain analysis [5,6]. Introduced in the 1990s to monitor anesthesia depth in humans [6,7,8], it has also been applied to animal species, including dogs [9], cats [10], rabbits [11,12,13,14,15], pigs [16,17], horses [18,19,20], goats [21], and chickens [22]. The Bispectral index ranges from 0 (cortical suppression) to 100 (awareness), and values between 40 and 60 have been considered reflecting an appropriate depth of anesthesia in humans [23]. No targets have been established in veterinary species and contrasting results have been found concerning its reliability [12,13,17]. Moreover, presence of artefacts and influence of different anesthetic drugs as well as interspecies differences in EEG features associated with different hypnotic states may also affect the correlation between the BIS value and depth of anesthesia.

In humans undergoing neurosurgical procedures, neuro-anesthesiologists aim to protect the brain from any alteration of its physiological function and to guarantee a controlled but rapid recovery from anesthesia to allow an early neurological assessment [24]. Evaluating BIS in these patients is considered difficult because a low value could reflect either drug-induced hypnosis, brain-injury, or both [25]. Moreover, modifications in cerebral blood supply can also occur when vessels are involved in the procedure, affecting BIS values [26]. The use of EEG monitors to evaluate changes in cerebral blood flow has been suggested to help preventing negative surgical outcome [27] and detecting intraoperative cerebral ischemia [28].

The number of rabbits undergoing general anesthesia, for both clinical and experimental reasons is increasing each year. In biomedical research, these animals often undergo neurovascular surgeries that may lead to an impairment in cerebral blood supply [29,30,31,32]. A non-invasive method to assess its occurrence could be of paramount importance to improve patient outcome and decrease morbidity in this species.

Nociception can provoke cortical arousal during general anesthesia, and this might lead to an increase in the BIS [10]. On the other side, the ability of antinociceptive drugs, in particular μ agonist opioids, to modify BIS is still controversial [17,33,34]. Indeed, while in humans some studies found that fentanyl increased the hypnotic effect of propofol without modifying the BIS [33,34], in anaesthetized pigs, fentanyl administration stabilized the BIS when raw EEG activation occurred [17].

No studies evaluating either the BIS usefulness and reliability or the effect of fentanyl on the BIS have been performed in a large cohort of rabbits during balanced anesthesia so far.

The primary aims of this study were (1) to evaluate whether a clinically adequate depth of anesthesia was mirrored by consistent and stable BIS values in rabbits undergoing neurovascular surgery and (2) to investigate whether BIS changes occur when cerebral blood supply is altered and restored through bilateral carotid clamping and re-opening, respectively.

The secondary aim was to investigate the effects of fentanyl, as antinociceptive drug, on the BIS.

We hypothesized that (1) the BIS would be a useful and reliable tool for the assessment of depth of anesthesia in rabbits undergoing neurovascular procedures, (2) changes in cerebral blood supply during carotid clamping would be reflected by the BIS and (3) fentanyl administration would diminish the BIS.

## 2. Materials and Methods

### 2.1. Study Design

This study was designed as prospective observational trial. Data were collected from animals used for another study aimed to develop a model of microsurgical carotid artery bifurcation aneurysms [29,30].

The study was reviewed and approved by the Committee for Animal Experiments of the Canton of Bern, Switzerland (Permission number: BE 108/16). For all experimental procedures the ARRIVE guidelines (Animals in Research: Reporting of In Vivo Experiments) were strictly followed.

### 2.2. Animals

Sixty-eight, healthy (ASA 1), females New Zealand White rabbits, a laboratory breed rabbits (*Oryctolagus cuniculus*), undergoing general anesthesia for surgical creation of carotid bifurcation aneurysms between March 2019 and March 2020 were enrolled.

The rabbits were housed in groups in the Central Animal Facility (CAF–Department for BioMedical Research, University of Bern). The facility guaranteed an adjusted climate (temperature 22–24 °C ± 2 °C, humidity 30–60% ± 5%) and special sun substitution with UV light (photoperiod 6–18 h). Water and pellet food was accessible ad libitum. Environmental enrichment was provided with hay and toys.

The morning of the surgery, rabbits were transferred using a transport box, where they could maintain visual and auditory contact with a co-mate, from the CAF to the experimental surgery facility (ESF), where anesthesia and surgery were carried out. At the end of the surgery, the rabbits were placed in individual recovery cages under constant veterinary supervision. When fully awake, rabbits were transferred back to the CAF, where they were single housed for 5–7 days.

### 2.3. Anesthetic Management

After pre-operative clinical assessment, the auricles of both outer ears were shaved and prilocaine- lidocaine cream (Anesderm 25 mg/g + 25 mg/g cream, Pierre Fabre SA, Allschwil, Switzerland) was applied to both the auricular arteries and veins. Afterwards, rabbits were sedated with a combination of ketamine 20 mg/kg (Narketan, Vetoquinol AG, Bern, Switzerland), dexmedetomidine 100 mcg/kg (Dexdomitor; Provet AG, Lyssach, Switzerland) and methadone 0.3 mg/kg (Methadon Streuli; Streuli Pharma AG, Switzerland), injected subcutaneously (SC) in the dorsal neck area. The animals were left undisturbed for at least 10 min in their transport box allocated in a dark and silent room. If the sedation was deemed inadequate (reaction to touch and manipulation) after 20 min, a supplementary dose of ketamine (up to 5 mg/kg) or dexmedetomidine (up to 5 mcg/kg) was administered; the exact top-up dose was chosen depending on the single animal need.

Once unresponsive to tactile and visual stimulation, the rabbits were moved on a table and positioned in sternal recumbency. Oxygen supplementation through a non-tight face mask (4 litres/minute) was started and continuously administered until tracheal intubation. Pulse rate (PR) and the oxygen blood saturation (SpO_2_) were monitored continuously through a pulse-oximeter probe positioned on a digit. A 22-gauge (G) cannula was inserted aseptically both into the right ear marginal auricular vein and into the left ear auricular artery. The surgical area was shaved and disinfected and ropivacaine 0.75%, 2 mg/kg (ROPIvacain Fresenius 7.5 mg/mL, Fresenius Kabi, AG, Switzerland) was injected intradermally in the peri-incisional area.

Propofol 1 mg/kg to effect (Propofol 1% MCT 200 mg/20 mL, Fresenius Kabi AG, Switzerland) and/or midazolam up to 1 mg/kg (Dormicum; Roche CH, Switzerland) were injected intravenously (IV) to allow tracheal intubation. Once intubated, the animals were transported to the operation room, positioned in dorsal recumbency and connected to the anesthesia machine (Primus^®^, Dräger) via a pediatric circle system. End-tidal isoflurane concentration (ETIso) was increased to maintain an adequate depth of anesthesia, which was assessed clinically during the entire procedure and considered adequate at Time Point 0 (TP0) when jaw tone, body movements, palpebral reflex and eye globe movements were absent, and in the interval between TP1and TP5 when also the response to toe pinch was absent. During maintenance, ETIso was maintained below the rabbit’s isoflurane minimum alveolar concentration (MAC) (2.05 ± 0.18%) and a maximal ETIso of 1.4% ± 0.1% was targeted. Lidocaine 50 mcg/kg/minute (Lidocaine 2%, Streuli Pharma AG, Switzerland) and fentanyl 5 mcg/kg/hour (Fentanyl 0.5 mg/10 mL, Sintetica, Bern, Switzerland) were administered as constant rate infusion (CRI) until the end of surgery. Spontaneous ventilation with permissive hypercapnia was allowed, but mechanical ventilation (SIMV) was initiated if partial pressure of carbon dioxide (PaCO_2_) exceeded 60 mmHg.

Heart rate, PR, RR, invasive arterial blood pressure, temperature, SpO_2_, BIS index and EMG, inhaled and End Tidal gas (O_2_, CO_2_ and inhalant agents) and ventilation parameters were continuously monitored and manually recorded every 5 min. The BIS parameters, i.e., BIS index, electromyography activity (EMG), burst suppression ratio (BSR) and signal quality index (SQI), were continuously registered on the central hard-disk via a multi-parameter monitor (Datex- Ohmeda S3, GE Healthcare Inc., Helsinki, Finland).

In case of hypotension, defined as mean arterial pressure MAP ≤ 60 mmHg or Doppler pressure ≤80 mmHg, noradrenaline 0.1–0.5 mcg/kg/min (Noradrenaline 1 mg/mL, Sintetica, Bern, Switzerland) was administered. Hypothermia (rectal temperature ≤ 38 °C) was prevented using a heating forced-air warming system (Mistral Air). Invasive arterial blood pressure was measured from the auricular artery until the left carotid artery was clamped. Afterwards, blood pressure was measured non-invasively with doppler technique, being the probe positioned at the level of pedal artery of the right foot. Ringer’s lactate at a continuous rate infusion of 5 mL/kg/h was administered during anesthesia.

At the end of the surgical procedure, the rabbits were positioned in lateral recumbency, weaned from the ventilator and trachea was extubated when the swallowing reflex was returned. Systemic analgesia was provided with meloxicam 0.5 mg/kg IV (Metacam, Boehringer Ingelheim Schweiz GmbH, Basel), antibiotic therapy with amoxicilline 20 mg/kg IV (Clamoxyl 20, GlaxoSmithKline Pharmaceutical s.a./n.v., Wavre) and supplemental therapies, such as acetylsalicylic acid 10 mg/kg IV (Aspégic Inject 0.5 g, Opella Healthcare AG, Schweiz) and 100 mcg of vitamin B12 (Vitarubin, cyanocobalamin 1000 mcg/mL, Streuli Pharma AG, Schweiz) SC were administered. Active warming and oxygen through a facial mask were supplied until the rabbits regained spontaneously sternal recumbency. Once fully recovered, a fentanyl patch 12 mcg/h was applied to the external auricular surface and kept in place for the following 72 h and the rabbits were transferred back to the CAF.

Each anesthetic procedure was carried out under the supervision of a board-certified veterinary anesthesiologist (DC).

### 2.4. Bis Positioning and Recording

**Figure 1:**
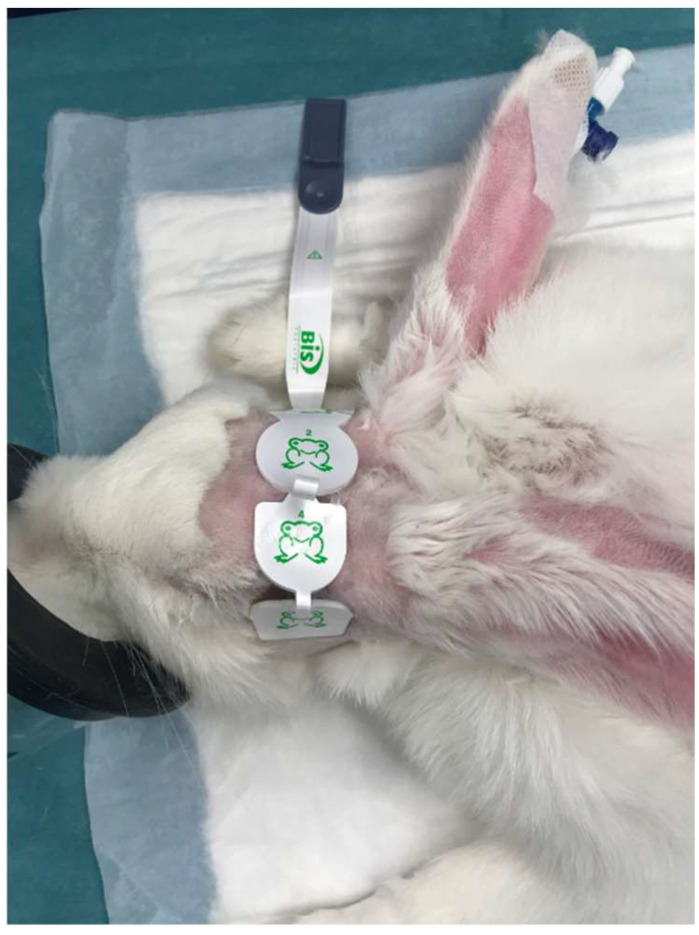
Positioning of the BIS pediatric sensor on the frontal bone in a rabbit.

After accurate clipping of the skull, the skin was defatted with benzinum medicinale for sensor placement. Subsequently, a BIS pediatric sensor (Medtronic Parkway Minneapolis, MN, USA) was placed on the frontal bone. Electrodes 1 and 3 were applied approximately 1 cm caudal to the lateral canthus of the right and left eyes, while electrodes 2 (ground) and 4 (floor) were applied on top of the frontal bone according to previous literature [11]. The BIS electrodes positioning was always performed by the same operator (DC) (Figure 1).

The BIS related parameters (EMG, BSR and SQI) and their ranges are reported in the Supplementary Materials (Figure S1).

**Figure 2:**
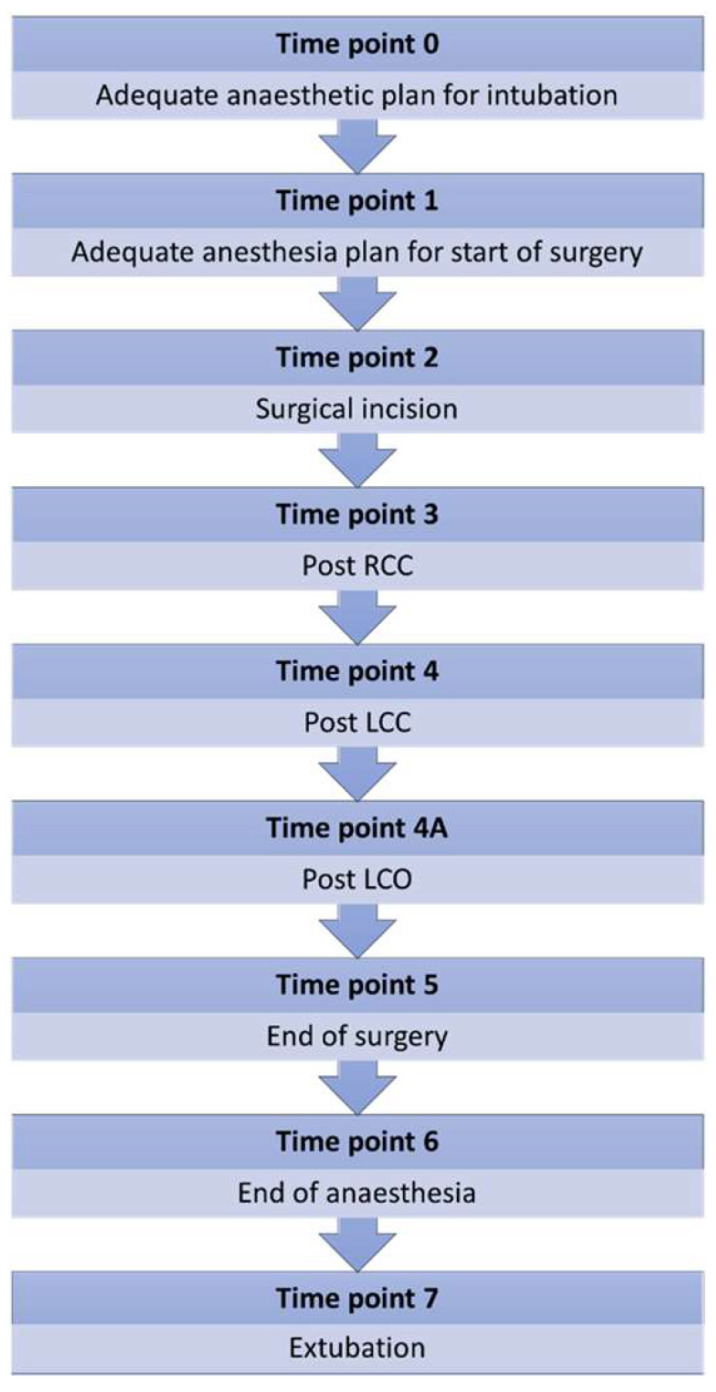
Schematic representation of the selected time points and their related events (TPs). RCC: right carotid closing; LCC: left carotid closing; LCO: left carotid opening.

Nine timepoints were chosen to evaluate the presence of variations in BIS and their potential association with changes of depth of anesthesia (clinically evaluated) and changes of cerebral blood supply (Figure 2):

- Time point 0 (TP0): depth of anesthesia deemed adequate to maintain the trachea intubated in absence of surgical stimuli.
- Time point 1 (TP1): depth of anesthesia deemed adequate to start the surgery.
- Time point 2 (TP2): surgical incision.
- Time point 3 (TP3): 1 min after right carotid clamping (RCC).
- Time point 4 (TP4): 1 min after left carotid clamping (LCC).
- Time point 4A (TP4A): 1 min after left carotid opening (LCO).
- Time point 5 (TP5): end of surgery.
- Time point 6 (TP6): end of anesthesia (ETIso 0.3%).
- Time point 7 (TP7): tracheal extubation.

The surgical procedure has been previously described [29,30]. Details are provided in the Supplementary Materials (Figure S2).

### 2.5. Antinociception

Intraoperative antinociception was considered insufficient if at least one parameter among heart rate, respiratory rate, and blood pressure increased by ≥20% compared to the baseline value, which was recorded in general anesthesia before surgical incision. In these cases, a fentanyl bolus of 5 mcg/kg IV was administered.

### 2.6. Statistical Analysis

Statistical analysis was performed using Microsoft Excel (Microsoft 365) and SigmaStat 4.0 (Point Richmond; CA, USA) software. Anesthesia protocols or experimental sheets with 50% missing data, and those in which more than 1.65% ETIso was reported at any point during general anesthesia, were excluded from further statistical analysis.

Data were assessed for normality with the Shapiro–Wilk test.

Normally distributed data are presented as mean ± standard deviation (SD); not normally distributed data are presented as median (1st quartile, 3rd quartile).

Data for ETIso, BIS, electromyography activity index (EMG), signal quality index (SQI), and burst suppression ratio (BSR) were recorded at 5 s intervals. For TPs 0, 1, 2, 3, 4, 4A, and 5, median values were calculated for the 180 s window following event onset, while for TP 6 and 7, median values were calculated for 60 seconds’ window. No statistical analysis was performed for ETIso at TP6 (as it was pre-set at 0.3%) and at TP7.

With the aim to evaluate BIS, EMG, SQI and BSR modifications related to rescue analgesia administration, median values were calculated for the 60 s following the nociceptive event (values indicated as “before”) and for the 60 s following fentanyl administration (values indicated as “after”). If two or more boli were administered within less than a 5 min interval, only the first was considered in the statistical analysis, to avoid bias linked to cumulative effect.

BIS changes after fentanyl administration were divided into 3 groups:

Increase: where the BIS value recorded after fentanyl administration increased ≥10% compared to the value recorded after the nociceptive event.

Decrease: where the BIS value recorded after fentanyl administration decreased ≥10% compared to the value recorded after the nociceptive event.

No modifications: where the BIS value recorded after fentanyl administration increased or decreased ≤10% compared to the value recorded after the nociceptive event.

Comparison between median and mean values recorded before and after an event, were performed using the Wilcoxon signed ranks test and the paired *t*-test, respectively.

Differences among TPs over time were assessed using the two-way repeated-measures analysis of variance (ANOVA) followed by the Tukey test.

The BIS values recorded at TP3 versus TP4, and at TP4 versus TP4A were compared with the Wilcoxon signed rank test, to assess if the clamping and re-opening of the carotid had an influence on this index.

Differences in BIS, EMG, SQI, and BSR before and after fentanyl bolus administration, were evaluated using the Wilcoxon signed ranks test.

Statistical significance was set at *p* < 0.05.

A post-hoc power analysis (Wilcoxon signed rank test; matched pairs) was performed (G*Power 3.1.9.4, Franz Faul, Universität Kiel, Germany), for which bispectral index values (means ± SD) at TP2 and TP6 were used, and the α error probability was set at 0.05.

## 3. Results

Data collected from fifty-nine female rabbits were included in the statistical analysis.

Mean age was 21.1 ± 1.1 months, mean weight was 3.7 ± 0.2 kg, and mean length of surgery was 222 ± 42 min.

Thirty-seven animals needed propofol to allow tracheal intubation, one animal received ketamine 1 mg/kg IV and one animal received midazolam 0.1 mg/kg IV after propofol induction. In twenty-two rabbits, the combination injected subcutaneously allowed tracheal intubation.

The maximal concentration of ETIso was 1.63% and it was reached at TP5.

**Figure 3:**
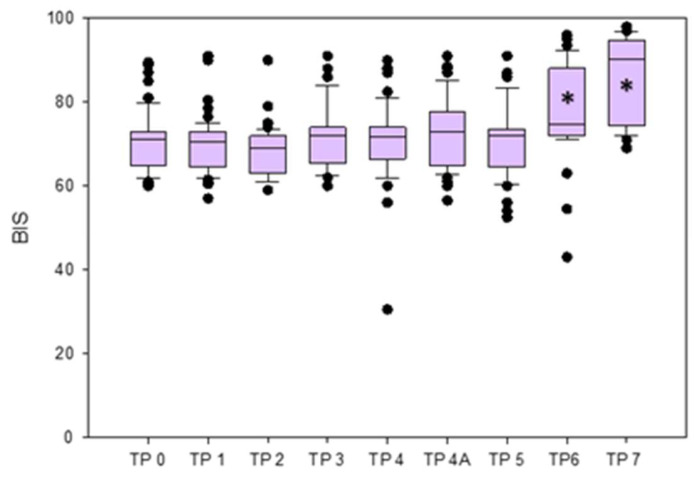
Box plots of BIS variations reported as median and interquartile range (25%,75%) in the different time points (TPs). * Statistically significant difference.

- The BIS was significantly lower at TPs 0, 1, 2, 3, 4, 4A, and 5 compared to TP7, and at TPs 0, 1, 2, 3, 4, 4A, and 5 compared to TP 6. No significant differences were found between TP3 (RCC) and TP4 (LCC), and between TP4 (LCC) and TP4A (LCO) when analyzed separately (Figure 3).
- The EMG values were significantly lower at TPs 0, 1, 2, 3, 4, 4A, 5, and 6 compared to TP7, and at TPs 2, 3, 4, 4A, and 5 compared to TP0. Values were significantly lower at TPs 3, 4, 4a, and 5 compared to TP6, while those at TPs 3, 4, 4A were significantly lower compared to TP1.
- The BSR remained 0 in all rabbits at all TPs, except for one rabbit that showed a sudden increase of BSR (value 44), accompanied by a BIS decrease, immediately after the LCC (TP4).
- The SQI results are reported in Supplementary Materials (Table S1).

Median and interquartile ranges (25, 75%) of ETIso, BIS, EMG, and BSR at the different time points and *p* values from the comparison between TPs are reported in Table 1.

**Figure 4:**
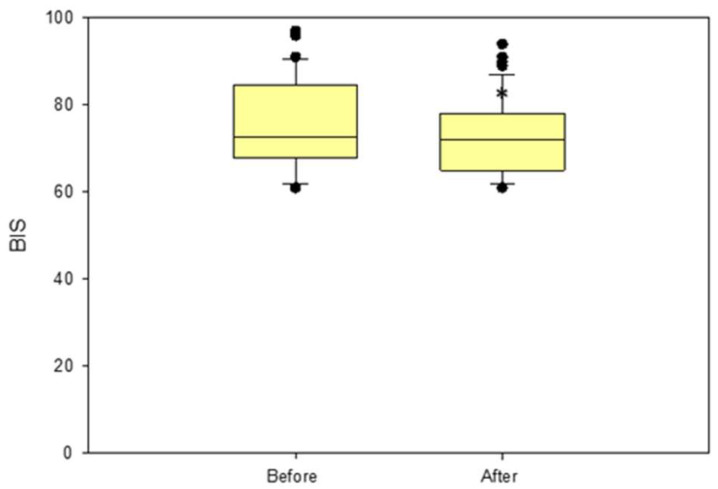
Box plots showing the effect of fentanyl bolus (5 mcg/kg IV) on the BIS, reported as median and interquartile range (25%, 75%) in all the rabbits. * Statistically significant difference.

**Figure 5:**
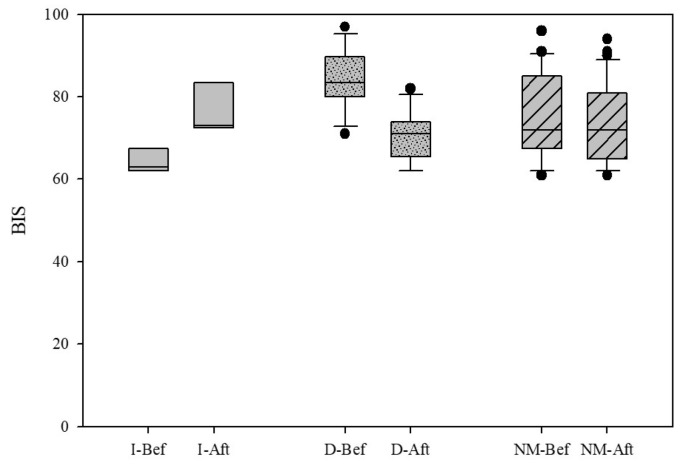
Box plots of the BIS values before (Bef) and after (Aft) fentanyl boli, in cases in which BIS values increase (I), BIS values decrease (D) and when no modifications (NM) in BIS values occurred. Values are reported as median and interquartile range (25%, 75%).

Forty-one animals received one or more rescue fentanyl boli, for a total of 62 boli administered. Values of BIS, EMG, SQI, and BSR before and after fentanyl administration are reported in Table 2. Overall, BIS (*p* = 0.018), but not the EMG and BSR, changed significantly after fentanyl administration (Figure 4). In particular, BIS showed three different trends following fentanyl administration: it increased its value in five cases, decreased in 12 cases, whereas no modifications were recorded in the remaining 44 cases (Figure 5).

Euthanasia was performed intraoperatively in one case due to surgical complications, and post operatively in two cases after recovery from anesthesia as the humane endpoint was reached. During the first week after the surgical procedure, spontaneous death occurred in two rabbits.

The post-hoc power analysis showed a power (1- β error) of 1.

## 4. Discussion

The results of the present study showed that during anesthesia BIS values were significantly lower than at recovery, and that BIS values were consistently comparable with the clinical evaluation of adequate depth of anesthesia. Furthermore, no BIS oscillations and no burst suppression in all rabbits except one were detected after carotid clamping. The administration of fentanyl, when signs of nociception were detected, decreased overall the BIS, but not in a clinically relevant way, and different trends among the rabbits were observed.

According to our results, BIS could be considered useful in helping clinicians to assess stability of depth of anesthesia in rabbits. Indeed, clinical adequate depth of anesthesia was accompanied by stable and consistent BIS values over time. Therefore, the lack of significant differences among the TPs 0 to 5, despite different ETIso values, is in line with our expectations and the study design. Indeed, at the beginning of anesthesia, the sedative drugs administered allowed an adequate anesthetic plane at lower isoflurane concentrations than at later stages. This finding is not surprising as combinations of full µ and α_2_ agonist with N-Methyl-D-aspartic Acid (NMDA) antagonists have been often reported to provide adequate anesthetic level in different species [35,36]. Furthermore, in the present study, they guaranteed sufficient depth of anesthesia for providing tracheal intubation in 22 out of 59 rabbits. As no studies investigating the effect of a ketamine, dexmedetomidine and methadone combination on BIS have been performed so far, neither in human nor in veterinary medicine, it is impossible to compare our results with others. Lidocaine has been previously shown to have both antinociceptive and MAC sparing effects in different animal species and in humans [37,38,39,40]. In our study, its use in combination with the other administered drugs, might have allowed to maintain a stable an anesthetic level with values of ETIso lower than MAC.

In this study, BIS values, indicative of a clinically adequate surgical anesthetic plane, were higher than those reported in human medicine (40–60) [23]. Moreover, EMG values were often high, indicating the presence of muscular activity, possibly influencing the BIS [41]. In a previous study in humans, lidocaine was shown to increase BIS values when combined with isoflurane during anesthesia maintenance [40]. Therefore, the use of lidocaine in our study might have contributed to the BIS values recorded. Furthermore, in a previous trial in rabbits anesthetized either with sevoflurane or propofol for abdominal surgery, higher BIS values were recorded in the sevoflurane group compared to the propofol group. In particular, values recorded 1 min after surgical incision ranged from 38–62 in the propofol group, whereas they ranged from 61–82 in the sevoflurane group [11]. Therefore, animals anesthetized with volatile anesthetic showed BIS values comparable to those of our study.

In the present study, except for one rabbit, no BIS oscillations and no BSR were detected after carotid clamping. This finding suggests that the change in cerebral blood supply induced by clamping of both carotids did not significantly affect the rabbits’ brain electrical activity, supposably due to the ability of the vertebral artery to take over cerebral blood supply.

As the BIS is based on the EEG activity, it might well be expected to reflect ischemic events [42]. According to this idea, Bonhomme et al. [43] investigated BIS variations in 36 patients undergoing carotid endarterectomy during propofol-remifentanyl anesthesia. They reported that BIS values increased in the 47% of the patients, decreased in the 25%, and remained unaltered (with values between 40 and 60) in the remaining 28% within the first 3 min of carotid cross clamping. This variability was confirmed in other studies [12,43,44,45] but no prospective studies investigating BIS were carried out in patients undergoing carotid endarterectomy in general anesthesia, therefore the scarce level of evidence makes any conclusion very difficult. In our study, no significant difference in BIS was found between time points corresponding to LCC and LCO. Indeed, BIS followed different trends, showing decrease, increase and unaltered values. However, although BIS modifications during cerebral ischemia were reported in human medicine [26,28,43], the primary goal of this index is not to detect changes in cerebral supply. In our specific case, we cannot exclude that a minor change in brain perfusion took place, and we did not measure the cerebral perfusion, but stable BIS and absence of raw EEG silence ruled out a severe alteration affecting the electrical activity.

In a previous report, Romanov et al. [12] described a paradoxical BIS increase and a decrease in signal quality index after the ischemic event in rabbits undergoing bilateral carotid clamping. Simultaneously, raw EEG silence occurred during cerebral ischemia, even though laser Doppler detected only moderate reduction in cerebral blood flow. The authors explained these findings either as a true reduction of cerebral blood flow or as an enhanced anesthetic related depression caused by brain blood flow redistribution linked to iatrogenic induced hypotension [12]. Previous studies in humans reported a progressive slowing of the raw EEG signal accompanied by a decrease in high-frequency activity and a generalized attenuation of voltage, leading to an isoelectric EEG line during prolonged ischemia [46]. In the present study, a thoughtful analysis of the raw EEG was not performed, however the raw EEG activity (1 channel) was observed during the whole procedure in all rabbits, and no burst suppression or flat line EEG were detected but in one case. This rabbit experienced a sudden decrease in BIS with concurrent increase in BSR immediately after the LCC. It lasted only few seconds as the artery was promptly re-opened and then clamped again more progressively. After the second clamping, the BIS did not change from its previous value. This occurrence could be explained in this particular rabbit by the inability of the vertebral artery to take over the brain perfusion in such a short time or that the ischemic event took place in a bigger area of the brain covered by the electrodes.

An anatomical study in rabbits described the arterial blood supply of the brain [47]. Blood is provided solely and equally by both the vertebral arteries (which join at the level of the foramen magnum to form the basilar artery) and the internal carotids. In particular, each internal carotid supplies the ipsilateral cerebral cortex with the exception of some occipital areas. However, because the internal carotid and the posterior cerebral arteries (a branch of the basilar artery) have anastomotic connections through the circle of Willis, it is not possible to delineate precise borders between their areas of distribution in the brain [47]. According to our findings, a temporary clamping of both common carotids did not compromise the cerebral blood circulation to the assessed brain areas; this could be related to the collateral circle, which help preventing reduction in cerebral blood supply. This assumption is supported by the fact that all the rabbits who survived the surgical procedure did not show any neurological impairment at the anesthesia recovery.

While interpreting BIS results, the influence of the EMG value needs to be taken into consideration. In our study, the EMG values ranged from a median of 40 (interquartile range: 38–41) during surgical anesthesia (from TP1 to TP5), to 49 (interquartile range: 47–52) during recovery. The EMG activity interference is recognized as one of the most important limitations of the BIS monitoring, as it can elevate its values [48]. Indeed, the EEG is recorded from surface electrodes that collect electrical signals not only from the brain, but also from the muscles and other sources of artefacts (noises arising from movements, electrical devices, and other ambient noises) [49,50,51]. As the EMG frequency (30–300 Hz) overlaps the EEG one (0.5–47 Hz for the BIS monitoring) [5] it can lead to an incorrect interpretation of the signal [48,52]. This drawback was highlighted in human medicine, when BIS is applied in critical intensive care unit patients, who are usually only sedated and not receiving neuromuscular blocking agents [52,53]. In our study, neuromuscular blocking agents were not administered, and despite a clinically adequate anesthetic level was achieved, high EMG activity was detected by the BIS. This could have been related to the surgical manipulations, very close to the head, external electrical interference (e.g., warming system [51]) or spontaneous muscle activity. However, due to the adequate depth of anesthesia achieved during all procedures, the latter option is less likely.

In our study, BIS changed significantly after fentanyl. However, as the difference in the median value was very small and did not appear clinically significant, we decided to evaluate BIS variation following fentanyl administration in every single rabbit. Through that, three different trends were recognized, showing that fentanyl could increase, decrease, or let BIS remain unaltered (when a 10% difference was used as cut-off) depending on the subject. This is in line with the still debatable role of opioids and nociceptive stimuli in modifying BIS [48]. We hypothesized that fentanyl would have reduced the BIS as antinociception has been recognized to have a key role in maintaining adequate depth of anesthesia, avoiding cortical arousal [1,10], but our results indicate a more individualized trend. Studies in ICU patients suggest a possible relation between BIS and nociception [54,55]. Coleman et al. described an increase of BIS in patients undergoing moderate to severe noxious thermal stimulations [54]. Brocas et al. 2001 demonstrated that BIS did not increase its value compared with control cases in ICU patients undergoing tracheal suction sedated with midazolam and fentanyl [55]. Guignard et al. reported that remifentanil did not decrease the BIS in absence of noxious stimulations, while it showed a dose-dependent decrease when laryngeal stimulation was applied [56]. These authors concluded that the BIS should be considered a measure of anesthesia depth, resulting from the balance between anesthetic depression and nociceptive arousal [56]. However, the complex changes nociceptive stimuli induce on the raw EEG could be incorrectly interpreted by index-based EEG monitors [57]. Further studies should be performed to investigate this field.

This study has some limitations that need to be acknowledged. Sample size was calculated for the main research project and not for the present study. However, the post-hoc analysis revealed a power of 1 for evaluating the reliability of the BIS in rabbits undergoing balanced anesthesia. For running this analysis, a time point reflecting a stable depth of anesthesia (TP2) and one reflecting light anesthesia (TP6) was chosen.

Despite a standardized positioning of the pediatric BIS sensor throughout the study, no validation of its use in rabbits has been reported so far. Surgery time and, consequently anesthesia time, was not equal for all animals, thus the time elapsing between clamping and opening of the carotids was not constant. In our study, purposefully, the anesthesia was maintained overtime at an adequate depth, so no information about reliability and usefulness of BIS in presence of different degree of anesthesia depth could be retrieved.

Furthermore, only female rabbits were included, while it is currently recommended to involve both sexes in biomedical research to minimize potential sex related bias of the study.

## 5. Conclusions

The BIS can help assessing stability of anesthesia depth in rabbits undergoing neurovascular surgery. Clinically adequate depth of anesthesia was mirrored by stable and consistent BIS values among rabbits with a standardized protocol of balanced anesthesia, but further studies using different anesthetic protocols need to be performed to confirm the present findings. Clamping of the carotids did not bring about isoelectric activity and change of the BIS but in one case; we think that the sensitivity of BIS in detecting changes in cerebral blood supply needs to be further investigated and correlated to a direct measurement of flow and perfusion pressure. The BIS can be modified by opioid administration, but the direction of change seems to be subjective.

## Figures and Tables

**Table 1:** Median and interquartile range (25%,75%) of end tidal isoflurane (ETIso), bispectral index (BIS), electromyographic activity (EMG), and burst suppression ratio (BSR) at the different time points (TPs). Significant difference of BIS and EMG between time points, and related *p* values, are reported.

Time Point	ETIso	BIS		EMG		BSR
TP0	0.49 (0.37–0.65)	71 (65–73)	TP0 vs. TP7 *p* < 0.001 TP0 vs. TP6 *p* < 0.001	43 (41.2–44)	TP0 vs. TP7 *p* = 0.037	0 (0–0)
TP1	0.76 (0.64–0.99)	70.5 (64.5–73)	TP1 vs. TP7 *p* < 0.001 TP1 vs. TP6 *p* < 0.001	42 (40.5–42)	TP1 vs. TP7 *p* < 0.001	0 (0–0)
TP2	0.93 (0.69–1.17)	69 (63–72)	TP2 vs. TP7 *p* < 0.001 TP2 vs. TP6 *p* < 0.001	41 (40–42)	TP2 vs. TP7 *p* < 0.001 TP2 vs. TP6 *p* = 0.029	0 (0–0)
TP3	1.29 (1.03–1.39)	72 (65.5–74)	TP3 vs. TP7 *p* < 0.001 TP3 vs. TP6 *p* < 0.001	39 (38–40)	TP3 vs. TP7 *p* < 0.001 TP3 vs. TP6 *p* < 0.001 TP3 vs. TP0 *p* < 0.001	0 (0–0)
TP4	1.36 (1.16–1.45)	71.7 (66.5–74)	TP4 vs. TP7 *p* < 0.001 TP4 vs. TP6 *p* < 0.001	38 (37–39)	TP4 vs. TP7 *p* < 0.001 TP3 vs. TP6 *p* < 0.001 TP4 vs. TP0 *p* < 0.001	0 (0–0)
TP4A	1.34 (1.28–1.41)	73 (64.7–77.7)	TP4A vs. TP6 *p* = 0.019	38 (36–40)	TP4A vs. TP7 *p* < 0.001 TP4A vs. TP6 *p* < 0.001 TP4 vs. TP0 *p* < 0.001	0 (0–0)
TP5	1.31 (1.27–1.39)	72 (64.5–73.6)	TP5 vs. TP7 *p* < 0.001 TP5 vs. TP6 *p* < 0.001	40 (38–44)	TP5 vs. TP7 *p* < 0.001 TP5 vs. TP6 *p* = 0.046 TP5 vs. TP0 *p* = 0.007	0 (0–0)
TP6	0.3	74.7 (72–88.1)		43 (40–47.2)	TP6 vs. TP7 *p* < 0.005	0 (0–0)
TP7	0	90.2 (74.5–94.7)		49 (47–52)		0 (0–0)

**Table 2:** Median and interquartile range (25%, 75%) of BIS, EMG, SQI, and BSR in rabbits before and after fentanyl bolus. ^A^ Statistically significant difference (*p* = 0.018).

Fentanyl Bolus (*n* = 61)	Before	After
BIS	73 (68.3–83.6)	72 (64.8–81.1) ^A^
EMG	39 (38–40)	39 (38–40)
SQI	95 (87.5–97)	95.5 (87–98.5)
BSR	0 (0–0)	0 (0–0)

## Data Availability

The data presented in this study are available upon reasonable request from the corresponding author (mariafrancesca.petrucci@unibe.ch).

## Supplementary Materials

The following supporting information can be downloaded at https://www.mdpi.com/article/10.3390/brainsci13020327/s1, Figure S1. Anaesthesia monitor during procedure. BIS and its related parameters are shown in the yellow box, Figure S2. The aortic arch (§) with its branches left (x) and right (#) carotid arteries (A). Ligatures representation on the proximal and distal right carotid artery (B). Harvested autologous arterial pouch (*) and the blunt of the right carotid artery sutured to the distal third of the left carotid artery (C). Artificial complex arterial bifurcation (D). This is adapted from Wanderer, S., Waltenspuel, C., Grüter, B. E., Strange, F., Sivanrupan, S., Remonda, L., Widmer, H. R., Casoni, D., Andereggen, L., Fandino, J., Marbacher, S. Arterial Pouch Microsurgical Bifurcation Aneurysm Model in the Rabbit. J. Vis. Exp. (159), e61157, doi:10.3791/61157 (2020) [1], Table S1. Median and interquartile range (25%,75%) of SQI at the different time points. Significant difference of SQI between time points, and related p values, are reported.
