# Supplementary material for: Usefulness and Reliability of the Bispectral Index during Balanced Anesthesia for Neurovascular Surgery in New Zealand White Rabbits
Source: Brain Sci. 2023 Feb 14;13(2):327. doi: 10.3390/brainsci13020327 (PMC9954492; doi:10.3390/brainsci13020327)
Supplement: Supplementary file 1 [file brainsci-13-00327-s001.zip › brainsci-2199635-supplementary.pdf]

Supplementary materials (S1)

1 Bispectral index related parameters

Electromyographic activity (EMG): this frequency range reflects power from muscle activity, as well as power from other high-frequency artifacts. It ranges from 0 to 100. During the procedures the EMG is shown as a power bar (see Figure 1). When the bar is empty, it indicates that EMG activity is low. The analysis of the related number can be done only after downloading the data.
Burst suppression ratio (BSR): EEG pattern characterised of alternating high amplitude bursts and isoelectric periods; it ranges from 0 to 100. On the monitor, its value is given as a number.
Signal quality index (SQI): indicates detected signal reliability and its value is calculated based on impedance and artefacts; it ranges from 0 to 100 (poor signal quality defined as SQI≤ 50). It is indicated by a power bar on the monitor. The analysis of the related number can be done only after downloading the data.
The anaesthesia monitor with BIS and its related parameters is shown in Figure S1.

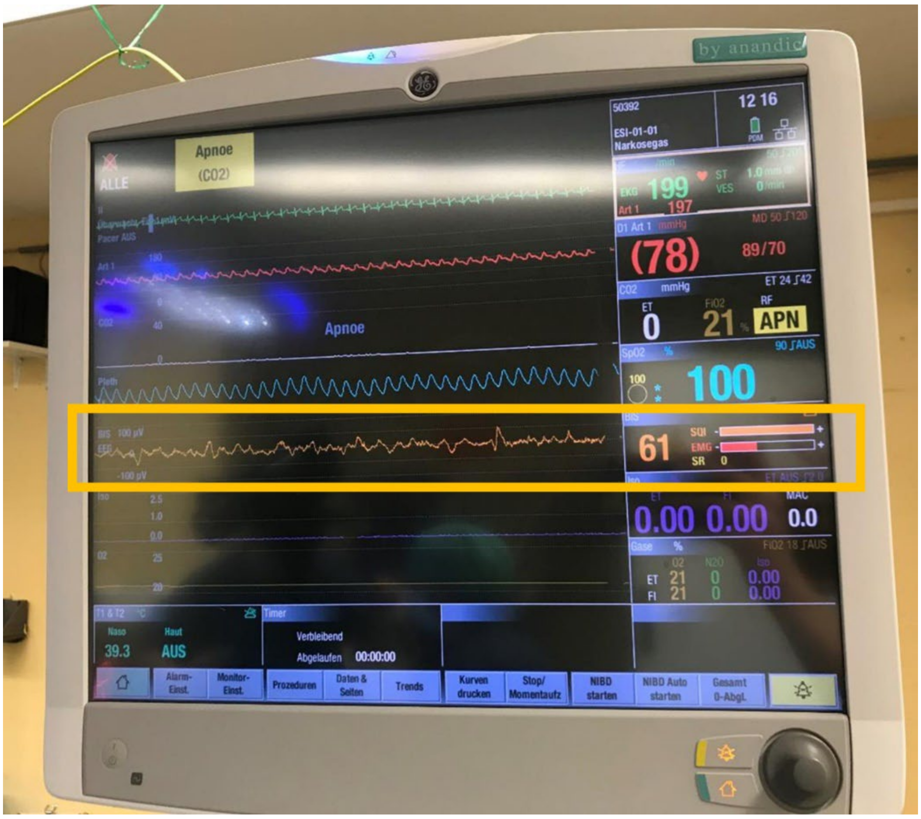

Figure S1. Anaesthesia monitor during procedure. BIS and its related parameters are shown in the yellow box.

2 Surgical procedure

Details about the methodology of the carotid bifurcation aneurysm model have been previously described[1,2]. A median skin incision from the manubrium sterni was performed to access the left common carotid artery (CCA). The artery was divided from the vagal nerve and papaverine was administered locally (Papaverin HCL 40 ng/ml, Bichsel AG, Interlaken, Switzerland); the same

procedure was subsequently performed on the right CCA. Before the ligation of the right proximal CCA, heparin was injected (500 IU/kg) systemically via a venous ear catheter. (Liquemin 25000 I.E/5 ml, Drossapharm, Arlesheim AG, Switzerland) (100 I.U./kg). Then, the right proximal CCA was ligated with a non-absorbable suture. Secondly, a non-absorbable ligature was applied 4–5 mm distally by using a vessel clip for measurement. The right CCA was clamped as far distally as possible with a temporary vessel clip (as normally used in cerebral aneurysm surgery) to avoid any endothelial damage and to create a long vessel segment for irrigation in order to prevent thrombogenesis.
The left CCA was subsequently clamped and an arteriotomy was performed. The arterial pouch previously collected was sutured on the left CCA and the blunt of the right carotid artery was sutured to the distal third of the left carotid artery (creating an artificial complex arterial bifurcation). Finally, the left carotid artery was de-clamped. At the end of the procedure, fluorescence angiography was performed and the restoration of the flow in the distal left CCA was confirmed through dying of the pouch and return of invasive arterial blood pressure curve (measured at the ear artery, a direct branch of the external carotid) [1]
A schematic representation of the procedure is shown in Figure S2.
In five animals, a slightly different procedure was carried out to pursue the same objective. Namely, a carotid artery side to end anastomosis was performed under parent artery patency with an endovascular balloon device. For suturing the bypass, the steps of the preparation were the same regarding separation of the left and right CCA as described above. A dry arteriotomy was performed after dissecting the femoral artery and entering it with a 4 French catheter. The endovascular device was applied by a neuroradiologist and inflated when radiographic markers were close to the arteriotomy side. Then, the right CCA was sutured end to side to the left CCA, the endovascular device deflated and removed. Femoral artery was successfully ligated, and the temporary clips removed.

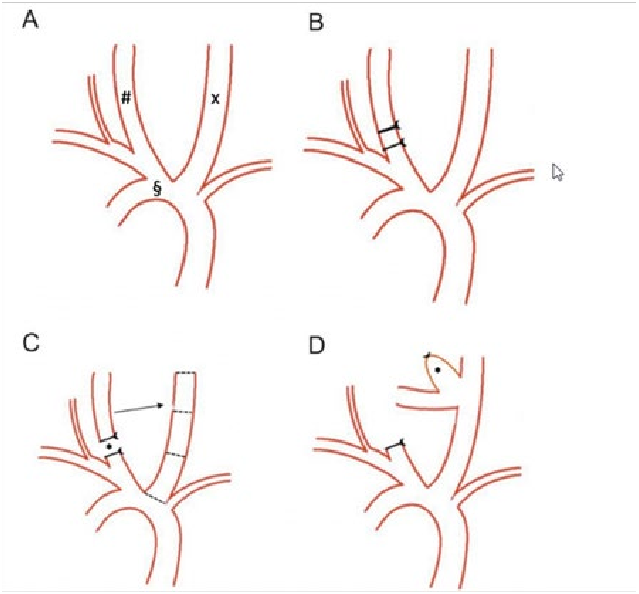

Figure S2. The aortic arch (§) with its branches left (x) and right (#) carotid arteries (A). Ligatures representation on the proximal and distal right carotid artery (B). Harvested autologous arterial pouch (*) and the blunt of the right carotid artery sutured to the distal third of the left carotid artery (C). Artificial complex arterial bifurcation (D). This is adapted from Wanderer, S., Waltenspuel, C., Grüter, B. E., Strange, F., Sivanrupan, S., Remonda, L., Widmer, H. R., Casoni, D., Andereggen, L., Fandino, J., Marbacher, S. Arterial Pouch Microsurgical Bifurcation Aneurysm Model in the Rabbit. J. Vis. Exp. (159), e61157, doi:10.3791/61157 (2020) [1].

3 Signal quality index (SQI) results

Table S1. Median and interquartile range (25%,75%) of SQI at the different time points. Significant difference of SQI between time points, and related p values, are reported.

Time point	SQI	SQI p value
TP0	97<br>(95- 100)	TP0 vs TP7<br>p<0.001<br>TP0 vs TP6<br>p<0.001<br>TP0 vs TP2<br>p=0.003
TP1	97<br>(94-100)	TP1 vs TP7<br>p<0.001<br>TP1 vs TP6<br>p<0.001<br>TP1 vs TP2<br>p=0.002
TP2	94<br>(86-97)	TP2 vs TP7<br>p=0.002<br>TP2 vs TP3<br>p=0.017
TP3	97<br>(95-97)	TP3 vs TP6<br>p=0.002<br>TP3 vs TP2<br>p=0.017
TP4	97<br>(94.3-97)	TP4 vs TP7<br>p<0.001<br>TP4 vs TP6<br>p<0.001<br>TP4 vs TP2<br>p=0.003
TP4A	97<br>(95-99.8)	TP4A vs TP7<br>p<0.001<br>TP4A vs TP6<br>p<0.001<br>TP4A vs TP2<br>p<0.001
TP5	94<br>(89.7-97)	TP5 vs TP7<br>p<0.001
TP6	92.75<br>(80.5- 96)	TP6 vs TP7<br>p=0.016<br>TP6 vs TP3<br>p=0.002
TP7	78<br>(72-85)	TP7 vs TP3<br>p<0.001

SQI: Values were significantly higher at time points (TPs) 0, 1, 4, 4A compared to TP7, at TPs 0, 1, 3, 4, 4A compared to TP6 and at TPs 0, 1, 3, 4 and 4A compared to TP2. Values were significantly lower at TPs 2, 7, and 6 compared to TP3. Values were significantly higher at TPs 2, 5 and 6 compared to TP7.

References

1. Wanderer, S.; Waltenspuel, C.; Grüter, B.E.; Strange, F.; Sivanrupan, S.; Remonda, L.; Widmer, H.R.; Casoni, D.; Andereggen, L.; Fandino, J.; et al. Arterial Pouch Microsurgical Bifurcation Aneurysm Model in the Rabbit. *J Vis Exp* 2020, doi:10.3791/61157.

2. Marbacher, S.; Erhardt, S.; Schläppi, J.-A.; Coluccia, D.; Remonda, L.; Fandino, J.; Sherif, C. Complex Bilobular, Bisaccular, and Broad-Neck Microsurgical Aneurysm Formation in the Rabbit Bifurcation Model for the Study of Upcoming Endovascular Techniques. *Am J Neuroradiol* 2011, *32*, 772–777, doi:10.3174/ajnr.a2374.
